# Meta analysis on the efficacy of pharmacotherapy versus placebo on anorexia nervosa

**DOI:** 10.1186/s40337-014-0027-x

**Published:** 2014-10-30

**Authors:** Jasmijn de Vos, Laura Houtzager, Georgia Katsaragaki, Elske van de Berg, Pim Cuijpers, Jack Dekker

**Affiliations:** Arkin Mental Health Institute, Klaprozenweg 111, PO Box 75848, 1070 AV Amsterdam, the Netherlands; De Viersprong Mental Health Institute, Bergen op Zoom, the Netherlands; Vrije Universiteit, Amsterdam, the Netherlands

**Keywords:** Pharmacotherapy, Placebo, Treatment, Anorexia nervosa, Meta-analysis

## Abstract

**Background:**

Anorexia Nervosa (AN) has a devastating impact on the psychological and physical well being of affected individuals. There is an extensive body of literature on interventions in AN, however more studies are needed to establish which form of pharmacotherapy is effective. The few meta-analyses that have been done are based on one type of medication only. This article is the first to present data on three different, most commonly used, forms of pharmacotherapy. The primary objective of this meta-analysis was to create an overview and to determine the efficacy of three forms of pharmacotherapy (antidepressants, antipsychotics, hormonal therapy) compared to treatment with placebo in patients with AN.

**Method:**

A systematic literature search was performed to identify all randomized controlled intervention trials investigating the effectiveness of pharmacotherapy for AN within the following databases: PubMed, PsycINFO, Embase and Cochrane Library. In addition, 32 relevant reviews and meta-analyses were screened for additional intervention studies. A meta-analysis was performed on a total of 18 included studies (N = 869). Efficacy was measured in terms of weight gain or weight restoration.

**Results:**

The pooled effect sizes indicating the difference between antidepressants and placebo, and between antipsychotics and placebo on weight were not significant. Because of the small sample size no meta regression and subgroup analyses could be conducted. The pooled effect size indicating the difference between hormonal therapy and the placebo condition on weight (all weight measures) at post-treatment was 0.42 (95% CI: 0.11 ~ 0.73), which was significant. For hormonal therapy heterogeneity was high (I^2^ = 64.70). No evidence for publication bias was found. Meta-regression analyses of the weeks of medication treatment (slope = −0.008) yielded a significant effect (p = 0.04).

**Conclusions:**

In this study we found that hormonal therapy has a significantly larger effect on weight compared to placebo in the treatment of AN. However for these analyses heterogeneity was high, which means that these results have to be regarded with caution. We found that anti-depressants and antipsychotics had no significant effect on weight compared to placebo in the treatment of AN, although the power to detect significant effects was too low.

**Electronic supplementary material:**

The online version of this article (doi:10.1186/s40337-014-0027-x) contains supplementary material, which is available to authorized users.

## Background

### Anorexia nervosa

Anorexia Nervosa (AN) has a devastating impact on the psychological and physical wellbeing of affected individuals. The disorder is characterized by restricting intake of calories and energy which will lead to a low body weight, extreme fear of gaining weight and being preoccupied with behavior that will avoid gaining weight, and is usually accompanied by a significant disturbance in the perception of the shape and or size of a person’s body. In addition, body shape and weight have an undue influence on the affected persons self-esteem and self-evaluation [1].

At least 90% of individuals with AN are female. The prevalence is 0,5% - 1,0%. The course and outcome of AN are highly variable. AN has a high comorbidity with depressive symptoms, anxiety and obsessive compulsive symptoms. For a great part the etiology remains to be discovered. However, it is clear that a combination of multiple factors, genetic, neurobiological as well as psychosocial, lead to the development of this disease [2].

Treatment goals in AN include restoration of normal body weight, treat physical complications, a normal and healthy eating pattern, improvement of body image and self-esteem. Equally important is the improvement of other co morbid psychological symptoms. Because AN is associated with other psychopathology the treatment is often long and may involve several stages and intervention types [2].

### Treatment

Pharmacotherapy is often used in the treatment of AN. The fact that pharmacological interventions are established forms of treatment of several disorders that overlap with AN, has led many to conclude that pharmacotherapy may be useful in symptom reduction in AN [3]. According to Claudino et al. the rationale for pharmacological treatment of AN is based on neurobiological research into the control of appetite and food intake and on biological models of AN, on clinical observations and uncontrolled studies [4] The focus of pharmacological interventions in AN depends on the phase of illness. In the acute phase drugs are given to increase body weight and reduce AN symptoms (such as recurring thoughts about weight, caloric intake, depression, anxiety and obsessive/compulsive symptoms). In the second phase pharmacotherapy is expected to improve underlying psychopathology and prevent relapse [4,5].

Various types of medication have been studied in the treatment of AN; antidepressants, antipsychotics, nutritional supplementation and hormonal medication [3,5-7]. Agras and Robinson conclude in their review that there are no evidence-based psycho-pharmacological treatments available for either adolescent or adult patients with AN [8]. Two recent meta-analyses have reported on the effectivity of antipsychotics on anorexia nervosa [9,10]. Two other meta-analyses have been published on the efficacy of antidepressants and estrogens preparations [4,11]. In the described meta analyses different outcome measures were used, for example not only weight, but also bone health [11]. The overlapping conclusion of these articles is that more research needs to been done and they recommend the use of similar outcome measures. In this article we have set a step in that direction. This is the first study to present meta analyses on three different, most commonly used, forms of pharmacotherapy (antidepressants, antipsychotics, hormonal therapy) and report on the primary outcome measure for anorexia nervosa; weight.

### Lack of evidence

Considering the abovementioned findings, the results of pharmacotherapy compared to placebo treatment are scarce. Despite a considerable number of trials performed to elucidate the efficacy of three different forms of pharmacotherapy on AN, it remains unclear how far the results can be attributed to placebo effects. This can be explained by a number of factors; many patients with AN are difficult to engage in medical treatment and are unwilling to participate in randomized controlled trials, and many of these patients are so ill that they require a multiplicity of interventions [5]. As Powers & Santana state surprisingly few studies have been undertaken for AN and more over few studies have actually evaluated medications know to cause weight gain [12].

Although there are several studies published on AN as a symptom of other diseases, for example cancer, we chose to focus on AN as an eating disorder and on randomized controlled trials with a placebo condition. There are various systematic reviews exploring pharmacotherapy for AN but as far as we know only four meta-analyses have been published on the subject; one meta-analysis by Claudino et al. [4] that examines the effectivity of antidepressants for AN, one that reports on the effects of estrogen preparations [11] and two on the efficacy on antipsychotics [9,10]. The present study adds to the available body of evidence by providing an overview of the three most commonly used medicament treatments for AN: antidepressants, antipsychotics and hormonal medication. The meta-analysis of Sim et al. [11] doesn’t report on weight as a primary outcome measure, but on bone density loss. They also included cohort studies with “no medication” control groups instead of placebos. Thus, the present meta-analysis is the first one to report on the effects of hormonal medication on weight restoration. Furthermore, this meta-analysis contains an up to date search; the meta-analyses on antidepressants and estrogen preparations [4,11] performed their searches until April 2005 and march 2008 respectively. We chose to focus on pharmacotherapy versus placebo conditions only, while previous meta-analyses have also included studies comparing pharmacotherapy with treatment-as-usual [9] and studies comparing pharmacotherapy with pharmacotherapy [4,10]. Finally, subgroup analyses were performed where possible; only two previous meta-analyses have performed subgroup analyses [9,10].

## Method

### Search strategy

This meta-analysis is part of broader meta-analysis project on eating disorders. An extensive electronic database search for open and randomized controlled trial (RCTs) was conducted within the following databases: PubMed, PsycINFO, Embase and Cochrane Library. The search terms (both text words and MeSH terms) included a wide range of combined terms indicative of eating disorders (e.g. anorexia nervosa, bulimia nervosa, binge eating disorder, eating disturbance) and therapy (e.g. psychotherapy, nutrition therapy, counselling). The Additional file 1 “PubMed search string” contains an example of the search terms used. The complete search terms and filters used are available on request from the corresponding author. The screening process consisted of a number of steps. During all screening phases, the references were rated by three independent researchers (JdV, GK, LH). Disagreements were discussed and resolved in consensus and in cases of unresolved disagreement a senior reviewer (JD) was consulted. Studies were then either included or excluded from further analysis. The first step consisted of the application of the inclusion criteria to the 9722 abstracts and titles. Studies were selected if they (a) reported an author/ had an abstract, (b) were about treatment of eating disorders, (c) were written in English or Dutch. In the second phase the database was split into three eating disorder groups (Anorexia Nervosa, Bulimia Nervosa and Binge Eating Disorder). In the third phase we screened 32 earlier reviews and meta-analyses concerning treatment of eating disorders for additional relevant studies.

### Selection of studies

The next step was to proceed with the anorexia nervosa studies. For this meta/analysis we focus on randomized controlled trials. A total of 139 potential anorexia RCTs remained for a subsequent full-text screening. The database was split into two forms of treatment, pharmacotherapy and psychotherapy. In this meta/analysis, we included studies if they were a (a) randomized controlled trial, and (b) comparing pharmacotherapy with an placebo controlled condition and reported on (c) patients with Anorexia Nervosa with an age minimum of 12 years. Outcome had to be measured in (d) terms of weight gain. Studies in the acute and maintenance phase of treatment were both included.

### Meta-analysis

We conducted meta-analyses on a) antidepressants, b) antipsychotics and c) hormonal medication. Those were the most commonly used pharmacological treatments. For all three we conducted a separate meta-analysis on weight, the primary measure, comparing pharmacotherapy versus placebo.

#### Primary outcome measures

Most randomized controlled trials report some kind of weight measure as primary outcome measures. Only published data were used. The selection of primary outcome measures for this meta-analyses includes a range of weight related variables:

Efficacy at the end of treatment, measured in terms of weight gain or weight restoration as follows:

-  post weight in kg: the kg of the experimental and control group at the end of treatment.
-  post weight in BMI. This is an index for weight in relationship with height, as reported at the end of treatment for both experimental and control group.
-  post weight in IBW. Weight measured in percentage of Ideal Body Weight.
-  weight gain in kg and g. The difference in weight from pre-treatment to post treatment, as reported at the end of treatment.
-  IBW gain. The difference in IBW from pre-treatment to post treatment, as reported at the end of treatment.
-  post lean body mass. The fat free mass of the experimental and control group at the end of treatment.
-  post % fat mass. The absolute amount of body fat of the experimental and control group at the end of treatment.

Effect sizes (Cohen’s d) were computed for each of the primary studies. The post- to post-pharmacotherapy effect sizes were calculated by subtracting the average post-treatment score of the pharmacotherapy condition from the average post-treatment score of the placebo condition and dividing the result by the pooled standard deviations of both conditions Effect sizes of 0 – 0.32 are considered to be small, whereas effect sizes of 0.33 – 0.55 are moderate, and effect sizes of 0.56 – 1.2 are large [13].

To calculate the pooled mean effect size, we used the statistical (computer) program Comprehensive Meta Analysis (version 2.2.021; Biostat, Englewood, NJ, USA). Only measures explicitly describing weight at post treatment were used. When means and standard deviations were not presented, we used other statistics (e.g. *t-*value, *p-*value) to compute the effect size (n = 4). When neither the means and standard deviations nor a statistical test between the relevant scores was presented, the study was excluded because the data were not suitable for this meta-analysis.

As an indicator of homogeneity, we calculated Q-statistics. A significant Q-value rejects the null hypothesis of homogeneity. We also calculated the I^2^ statistic, which is an indicator of heterogeneity in percentages. A low value of 0% indicates that there is no observed heterogeneity, higher percentages indicate increasing amounts of heterogeneity, of which the highest is a value of 75% which indicates a high amount of heterogeneity (31).

Publication bias was tested according to Duval and Tweedie's trim and fill procedure [14] using Comprehensive Meta-analysis. We ran the publication bias analyses on all primary outcome measures.

### Subgroup analyses

Subgroup analyses were performed using the procedures implemented in Comprehensive Meta-analysis (version 2.2.021; Biostat, Englewood, NJ, USA). In this subgroup-analysis, studies were divided into two or more subgroups. For each subgroup the pooled mean effect size was calculated, and a test was conducted to examine whether the subgroups' effect sizes differ significantly from one another. We used the mixed effect model of subgroup-analyses, which pools studies within subgroups with the random effects model, but tests for significant differences between subgroups with the fixed effects model.

We conducted subgroup analyses for the following characteristics:

- Mono treatment (only pharmacotherapy or not)
- Setting: inpatient or outpatient or other (not reported or a combination of in-and outpatient)

The risk of bias quality assessments were based on the domains described by the Cochrane Collaboration [15]. The domains were evaluated for each study by two independent researchers. A code was given for each domain: yes (=the study reports correctly on this domain and there is no risk of bias), no (=the study reports on this domain but according to the description the domain could be biased) and unclear (=the study does not provide sufficient information to make an assessment). Disagreements were discussed and resolved in consensus and in cases of unresolved disagreement a senior reviewer (JD) was consulted. The domains that assessed the risk of bias are the following:

- Sequence generation: describes whether there was a random component in sequence generation
- Allocation concealment. describes whether assignment could be foreseen
- Blinding. Were participants and/or personal blind for the treatment condition
- Incomplete data. Did the study report on missing outcome data
- Selective outcome. Were all expected outcomes reported
- Risk of bias. Assesses total risk of bias

Meta regression analyses were performed in order to assess whether pre-treatment mean weight (kg and BMI), mean age, duration of illness (in months) and *n* weeks predicted the effect sizes. A significant positive or negative slope suggests that the variable is associated with the outcome.

### Power calculation

Because we expected only a limited number of studies, we conducted a power calculation to examine how many studies would have to be included in order to have sufficient statistical power to identify relevant effects. We conducted a power calculation according to the procedures described by Borenstein and colleagues [16]. We hoped to find a sufficient number of studies to be able to identify a small effect size of 0.3. These calculations indicated that we would need to include at least 20 studies with a mean sample size of 30 (15 participants per condition), to be able to detect an effect size of *d* = 0.30 (conservatively assuming a medium level of between-study variance, τ2, a statistical power of 0.80, and a significance level, alpha, of 0.05). Alternatively, we would need 15 studies with 40 participants each to detect an effect size of *d* = 0.30, or 14 studies with 50 participants.

## Results

### Inclusion of studies

**Figure 1:**
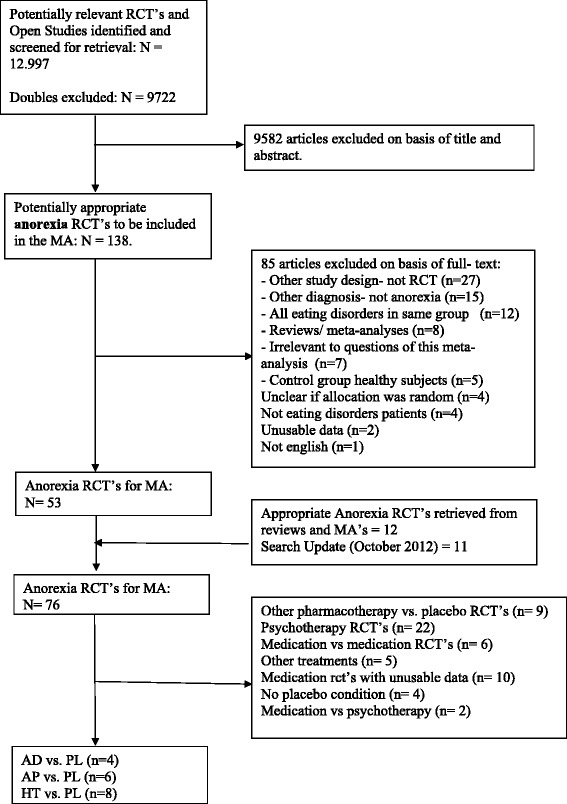
**Flowchart of the search process.**

The search yielded 12.997 results: PubMed (3675), PsycINFO (3660), Embase.com (5382) and Cochrane Library (280). The latest search update was performed in October 2012. After the duplicates were removed, 9722 titles and abstracts remained. A flow-chart showing the progress of the study selection is provided in Figure 1. After the last screening of full-text articles the search resulted in a total of 18 studies [17-34], which compared antidepressants, antipsychotics or hormones with placebo (Table 1 shows the treatment conditions for each study).

**Table 1:** **Characteristics of the included studies**

**1st Author, Year**	**Length of RCT**	**Recruitment**	**Anorexia type**	**Treatment conditions**	**Baseline mean weight** ^**a**^	**N**	**Mean age**	**Type and dosage** ^**b**^	**Setting**	**Adjunctive treatment**	**Weight outcome measures**	**Drop-out**
Attia, 1998 [17]	7 weeks	Clinical	Restricting/Binge-purge	1. AD	73 (IBW)	15	26	Fluoxetine 60 mg	Inpatient	1. Individual therapy	1.% IBW	27%
				2. Placebo	72 (IBW)	16				2. Group therapy	2. Change in % IBW/day	25%
										3. Family therapy		
										4. Behavior therapy		
										5. Caloric repletion		
Attia, 2011 [18]	8 weeks	Clinical	Restricting/Binge-purge	1. AP	16.7	11 12 (1 male)	27.7	Olanzapine	Outpatient		1. BMI	26%
				2. Placebo	17.4			2.5 mg				25%
								5 mg				
								10 mg				
Bissada, 2008 [19]	10 weeks	Clinical	Restricting/Binge-purge	1. AP	16.39	18	29.7	Olanzapine	Day hospital	1. Meal supervision	1. BMI	14%
				2. Placebo	15.93	16	23.6	2.5 mg		2. Group therapy		28%
								5 mg				
								7.5 mg				
								10 mg				
Brambilla 2007a [20]	3 months	Clinical	Unknown	1. AP	15.5	15	23.7	Olanzapine	Outpatient	1. CBT	1. BMI	14%
				2. Placebo	15.8	15	26.3	2.5 mg				
								5 mg				
Brambilla 2007b [21]	3 months	Clinical	Restricting/Binge-purge	1. AP	15.7	10	23	Olanzapine	Outpatient	1. CBT	1. BMI	n.m.
				2. Placebo	16.3	10		2.5 mg		2. Nutritional rehabilitation		
								5 mg				
Di Vasta 2012 [22]	18 months	Clinical	Restricting/Binge-purge	1. H	18.1	47	18.1	Dehydroepiandrosterone	Outpatient	1. Routine care = medical, nutritional, psychological monitoring	1. Kg	34%
				2. Placebo		47		50 mg			2. BMI	38%
											3. Lean mass	
											4. Fat mass	
**1st Author, Year**	**Length of RCT**	**Recruitment**	**Anorexia type**	**Treatment conditions**	**Baseline mean weight** ^**a**^	**N**	**Age**	**Type**	**Setting**	**Adjunctive treatments**	**Weight outcome measures**	**Drop-out**
Fazeli 2010 [23]	12 weeks	Community and clinical	Restricting/Binge-purge	1. GH	17.4	10	28	Nutropin	Outpatient		1. Kg ch.	10%
				2. Placebo	17.2	11	29.2	15 mg			2.%IBW ch.	18%
											3.% lean ch.	
											4. Lean ch.	
											5. Extremity lean ch.	
											6.% fat ch.	
											7. Fat mass ch.	
											8. Trunk fat ch.	
											9. Extremity fat ch.	
Grinspoon 1996 [24]	9 months	Clinical	Restricting/ Binge-purge	1. GH 30 mg	16.3	11	23	Insulin-like growth factor I	Inpatient		1.%IBW	0
				2. GH 100 mg	17	11		30 or 100 mg			2. BMI	19%
				3. Placebo	15.6	11						9%
Grinspoon 2002 [25]	6 days	Clinical	Restricting/ Binge-purge	1. GH + H	35 kg	16	24.2	Recombinant human IGF-I + Ovcon	Outpatient	1. Calcium	1.Kg	12%
				2. GH	35 kg	14	23	2x 30 mg		2. Multivitamins	2. Lean body mass (kg)	28%
				3. Placebo + GH	35.4 kg	15	27.6					0
				3. Placebo	32.3 kg	15	26.3					7%
Halmi 1986 [26]	32-45 days	Clinical	Restricting/ Binge-purge	1. AH	79 (IBW)	23	20.56	Amitryptyline	Inpatient		1. Days to target weight	n.m.
				2. AD	77 (IBW)	24		160 mg (max)			2. Average kg gain/day	
				3. Placebo	75 (IBW)	25						
Hill 2000 [27]	28 days	Clinical	Restricting/ Binge-purge	1.GH	14	7	14.5	Recombinant human growth hormone	Inpatient	1. Standard treatment	1. Average kg gain/day	n.m.
				2. Placebo	15	8 (1 male)	15	0.05 mg				
**1st Author, Year**	**Length of RCT**	**Recruitment**	**Anorexia type**	**Treatment conditions**	**Baseline mean weight** ^**a**^	**N**	**Age**	**Type**	**Setting**	**Adjunctive treatments**	**Weight outcome measures**	**Drop-out**
Kafantaris 2011 [28]	10 weeks	Clinical	Restricting	1. AP	16.9	10	17.1	Olanzapine	Outpatient and inpatient	1. Individual medication	1. BMI	30%
				2. Placebo	16	10		2.5 mg		2. Psychotherapy	2.%MBW	20%
								5 mg		3. Family therapy		
								7.5 mg		4. Nutritional therapy		
								10 mg				
Lacey 1980 [29]	n.m.	Clinical	Restricting/ Binge-purge	1. AD	40.6 (kg)	8	n.m.	Clomipramine	Inpatient	1. Individual psychotherapy	1. Kg	25%
				2. Placebo	37.7 (kg)	8		50 mg		2. Caloric repletion	2. Mean kg gain	12%
											3. Rate kg gain/day	
Miller 2011 [30]	12 months	Community and clinical	Restricting/ Binge-purge	1. OM/ H	17.8	20	25.2	Risedronate/Testosterone	Outpatient		1. Kg	23%
				2. OM	17.6	20	25.3	35 mg/ 150 mg			2. Lean body mass	
				3. H	17.5	19	27.1					
				4. Placebo	17.9	18	26.9					
Misra 2011 [31]	18 months	Community and clinical	Restricting/ Binge-purge	1. H	17.4	55	16.5	Fysiologic estrogen replacement	Outpatient	1. Behavior therapy	1. Kg	56%
				2. Placebo		55		100 mg			2. BMI	54%
											3. Fat mass	
											4. Lean mass	
Strokosch 2006 [32]	13x28 days	Clinical	Restricting/ Binge-purge	1. H	17.9	61	15.2	Norgestimate/ Ethinyl Estradiol	Outpatient		1. Kg	34%
				2. Placebo	17.6	62	15.1	180-250 mg/ 35 mg			2. BMI	21%
Vandereycken 1984 [33]	2x3 weeks	Clinical	Restricting/ Binge-purge	1. AP	40.4 (kg)	9	23.2	Sulpiride	Inpatient	1. “Uniform therapeutic programme”	1. Daily g change	n.m.
				2. Placebo	38.3 (kg)	9	23.7	300 or 400 mg				
Walsh 2006 [34]	1 year	Community and clinical	Restricting/ Binge-purge	1. AD	15.4	49	22.4	Fluoxetine	Outpatient	1. CBT	1. BMI	57%
				2. Placebo		44	24.2	20 mg to 60 mg		2. Family therapy		57%
										3. Medication monitoring		

*Note.* AD = Antidepressants; AH = Antihistamines; AP = Antipsychotics; ch. = change; CBT = Cognitive Behavioral Therapy; GH = Growth hormone; GI = Gastrointestinal; H = Hormones (other); n.m. = not mentioned; OM = Osteoporosis medication.

^a^Weight is BMI unless stated otherwise.

^b^More than one dosage mentioned means an increase of dosage after X weeks.

### Study characteristics

All 18 studies were randomized controlled trials, reporting on a total of 869 subjects. The control conditions included 438 subjects and the experimental conditions of 431 subjects. Table 1 presents the study characteristics of these studies.

The majority of the studies (N = 12) included adult patients, two reported on adolescents and three reported on both adults-adolescents. The number of patients in experimental conditions ranged from 7 to 55 per study. Fourteen studies recruited participants from clinical populations. In the remaining four studies a combination of clinica l and community recruitment was used. The larger part of the studies included patients with restricted AN and the binging/purging variant (N = 16), one study reported on the restricting type only and for one study it was unknown. Mean pre-treatment weight score ranged from 14 to 18.1 (BMI). This indicates that patients were severely underweight. A normal, healthy BMI ranges from 20 to 25. Six studies reported on inpatients, ten over outpatients, one of patients in a day care program and one unknown. Only two studies had male patients.

The majority of studies reported on hormonal treatments (the comparisons were N = 10), six reported on antipsychotics and four reported on the use of antidepressants. A broad variety of adjunctive treatments were mentioned, such as individual therapy, group therapy, family therapy, behaviour therapy, caloric repletion, meal supervision, behavioural incentives and more.

The quality of the 18 included studies was not optimal. Although all studies were randomized controlled trials, only five studies were assessed as having a low risk of bias based on the Cochrane domains [15]. The rest of the studies were assessed as having a high risk of bias. Sixteen trials reported blinding for the assessors and/or the patients and for two it was unknown. A random component in the sequence generation was reported in only nine studies; the rest of the studies used a non- random approach (N = 2) or did not report it (N = 7). Allocation concealment was done properly in only seven studies and with a high risk of bias in eleven studies. We also evaluated the use of incomplete data focusing on whether or not intention-to-treat analyses were performed. Incomplete data were adequately imputed in ten cases. Two studies performed completers- only analyses and six did not report it. Finally, fourteen studies reported all their outcomes, two studies reported only significant outcomes and for two studies it was unclear. Table 2 presents the quality assessment per study.

**Table 2:** **Risk of bias assessments**

**Study**	**Sequence generation**	**Allocation concealment**	**Blinding**	**Incomplete data**	**Selective outcome**	**Total risk of bias assessment**
Attia, 1998 [17]	No	No	Yes	Yes	Yes	High
Attia, 2011 [18]	Yes	Yes	Yes	Yes	Yes	Low
Bissada, 2008 [19]	Yes	Yes	Yes	No	Yes	Unclear
Brambilla, 2007a [20]	Unclear	Unclear	Yes	Unclear	Yes	Unclear
Brambilla, 2007b [21]	Unclear	Unclear	Yes	Yes	Yes	Unclear
Di Vasta 2012 [22]	Yes	Yes	Yes	Yes	Yes	Low
Fazeli 2010 [23]	Unclear	Unclear	Unclear	Unclear	Yes	Unclear
Grinspoon 1996a [24]	Unclear	Unclear	Yes	Unclear	Unclear	High
Grinspoon, 1996b [24]	Unclear	Unclear	Yes	Unclear	Unclear	High
Grinspoon, 2002a [25]	Yes	Unclear	Yes	Yes	Yes	Unclear
Grinspoon, 2002b [25]	Yes	Unclear	Yes	Yes	Yes	Unclear
Halmi 1986 [26]	Unclear	Unclear	Yes	Unclear	No	Unclear
Hill 2000 [27]	Yes	Unclear	Yes	Unclear	Unclear	Unclear
Kafantaris 2011 [28]	Yes	Yes	Yes	Yes	Yes	Low
Lacey 1980 [29]	No	Unclear	Yes	Yes	Yes	Unclear
Miller 2011 [30]	Unclear	Unclear	Yes	No	No	Unclear
Misra 2011 [31]	Yes	Unclear	Yes	Yes	Yes	Low
Strokosch 2006 [32]	Yes	Unclear	Yes	No	Yes	Unclear
Vandereycken 1984 [33]	Unclear	Unclear	Yes	Unclear	Yes	Unclear
Walsh 2006 [34]	Yes	Yes	Yes	Yes	Yes	Low

*Note.* yes (=the study reports correctly on this domain and there is no risk of bias); no (=the study reports on this domain but according to the description the domain could be biased); unclear (=the study does not provide sufficient information to make an assessment).

Weight was assessed in various ways. We reported on combined ES. To assure that this did not influence the results we performed the meta/analyses including for example only BMI or kg. This did not have an effect on the results, we therefore report on all weights. Some studies had weight measures such as weight gain and IBW gain [17,23,26,27,29,33]. To ensure that this was a valid approach we looked at the equivalence of weight measures of the groups at baseline. Almost all studies (except Halmi, 1986) mentioned explicitly that there were no significant differences in weight at time of randomization.

### Pharmacotherapy versus placebo

**Figure 2:**
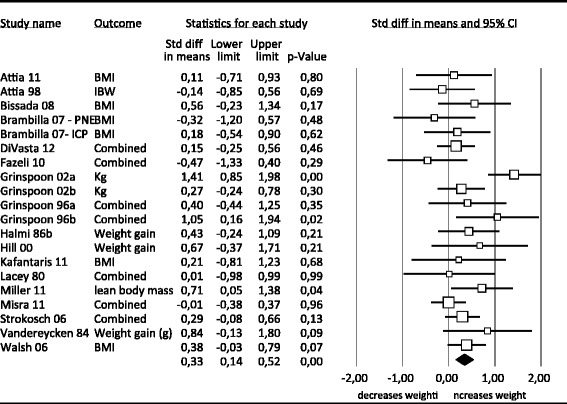
**Effects of pharmacotherapy vs. placebo on weight.**

Before performing the three meta-analyses of the most commonly used pharmacotherapy, we performed a meta-analysis comparing all three forms of pharmacotherapy with placebo. There were 20 studies that reported outcomes on weight. Figure 2 presents the outcomes of the meta-analysis.

The pooled effect size at post-treatment was 0.33, (95% CI: 0.14 ~ 0.52), indicating a significant effect. Heterogeneity was medium/ high (I^2^ = 40.08). There was one outlier [25]; when removed the effect size decreased and became 0.25, still remaining significant. When the outlier was removed, heterogeneity became low (I^2^ = 0). There was no evidence of publication bias. Subgroup analyses yielded no significant differences between subgroups. Meta regression analyses for mean age (slope = 0.014), duration of illness (slope = 0.014), mean weeks of treatment (slope = −0.0016) and mean BMI at the beginning of treatment (slope = 0.040) yielded no significant associations (*p* = 0.36; *p* = 0.89; *p* = 0.54; *p* = 0.50 respectively).

### Antidepressants versus placebo

**Figure 3:**
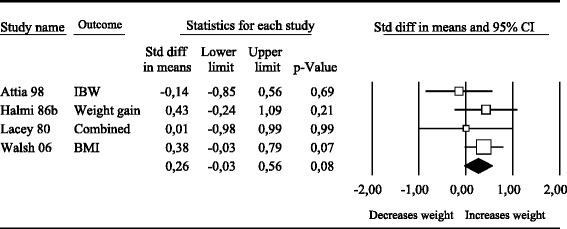
**Effects of antidepressants vs. placebo on weight.**

There were 4 studies that reported outcomes on weight (see Figure 3). The pooled effect size indicating the difference between antidepressants and the placebo condition on weight (all weight measures) at post-treatment was 0.26 (95% CI: −0.03 ~ 0.56), which was not significant (Table 3).

**Table 3:** **Meta-analyses of studies examining the effects of pharmacotherapy versus placebo for anorexia nervosa**

	***N***	***d***	**95% CI**	***Z***	***p***	***Q***	***I*** ^***2***^
***Pharmacotherapy vs. placebo***	20	0.33	0.14 ~ 0.52	3.47	0.00	31.71	40.08
**Outlier removed**	19	0.25	0.11 ~ 0.39	3.45	0.00	16.28	0.00
***Antidepressants vs. placebo***	4	0.26	−0.04 ~ 0.56	1.73	0.08	2.11	0.00
***Antipsychotics vs. placebo***	6	0.25	−0.09 ~ 0.60	1.43	0.15	3.74	0.00
***Hormonal treatment vs. placebo***							
**All studies**	10	0.42	0.11 ~ 0.73	2.67	0.01	25.50	64.70
**Outlier removed**	9	0.26	0.04 ~ 0.47	2.37	0.02	10.41	23.15
**One effect size per study (highest excluded)**	8	0.21	0.02 ~ 0.39	2.19	0.03	7.09	1.26
**One effect size per study (lowest excluded)**	8	0.45	0.07 ~ 0.83	2.31	0.02	25.39	72.43

The heterogeneity for Antidepressants was low (I^2^ = 0). The effect size comparing antidepressants with placebo at post-treatment was higher when adjusted for publication bias (d = 0.35; 95% CI: 0.06 ~ 0.64; number of trimmed studies = 1, right of mean), indicating a significant effect.

Because of the small sample size no meta regression or subgroup analyses could be conducted.

### Antipsychotics versus placebo

**Figure 4:**
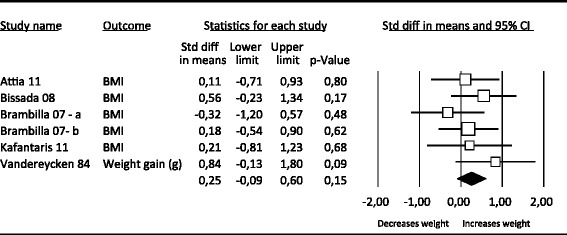
**Effects of antipsychotics vs. placebo on weight.**

Figure 4 shows that there were 6 studies that reported the effects of antipsychotics on weight. The pooled effect size indicating the difference between antipsychotics and the placebo condition on weight (all weight measures) at post-treatment was 0.25 (95% CI: −0.09 ~ 0.60), which was not significant (Table 3).

The heterogeneity for Antipsychotics was low (I^2^ = 0.00). No evidence for publication bias was found. Because of the small sample size no meta regression and subgroup analyses could be done.

### Hormonal pharmacotherapy versus placebo

**Figure 5:**
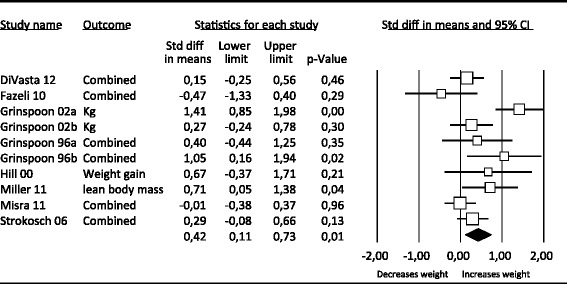
**Effects of hormonal medication vs. placebo on weight.**

There were 8 studies that reported on weight and 10 comparisons because of multiple conditions in some studies [24,25]; see Figure 5). Grinspoon et al. [24] administered 30 mg and 100 mg of recombinant human insulin-like growth factor I. In Grinspoon et al. [25] the therapeutic conditions consisted of recombinant human IGF-I and oral contraceptive administration. The pooled effect size indicating the difference between hormonal therapy and the placebo condition on weight (all weight measures) at post-treatment was 0.42 (95% CI: 0.11 ~ 0.73), which was significant (Table 3).

For hormonal therapy heterogeneity was high (I^2^ = 64.70). No evidence for publication bias was found. There was one outlier [25]; when removed the effect size decreased and became 0.26, still remaining significant. When the outlier was removed, heterogeneity became a bit lower (I^2^ = 23.15). Meta-regression analyses of the weeks of medication treatment (slope = −0.008) yielded a significant effect (p = 0.04). Meta regression analyses of BMI at the beginning of treatment (slope = −0.02326) and mean age (slope = 0.03932) yielded no significant effects (*p* = 0.83; *p* = 0.05 respectively). Subgroup analyses for hormonal therapy did not yield any significant results.

In this meta-analysis we included two studies in which two hormonal treatments were compared with the same control group [24,25], thus resulting in multiple comparisons in the same analysis. Because those comparisons are not independent from each other, this may have resulted in an artificial reduction of heterogeneity and have influenced the pooled effect size. We therefore performed a sensitivity analysis by including only one effect size per study. First, we conducted the analysis including only the comparison with the largest effect size from that study and then we did the same with the smallest effect size. As can be seen in Table 3, excluding the highest effect sizes resulted to a smaller (still significant) effect size and a large reduction in heterogeneity (I^2^ = 1.26).

## Discussion and conclusion

### Main findings

To our knowledge this is the first meta analysis focusing on three forms of pharmacotherapy in the treatment of AN. By analyzing these data we aimed to provide an overview of more detailed information of the effectiveness of pharmacological interventions on AN, compared to placebo.

When grouping all medication together, we found that pharmacotherapy is more effective than placebo. This grouping allowed an increase in power in order to perform subgroup and meta regression analyses. Unfortunately, they did not yield significant results. When performing meta-analyses for the three most common medicine apart, we found that hormonal therapy has a significantly larger effect on weight compared to placebo in the treatment of AN. This is a moderate effect size [13]. However for these analyses heterogeneity was high, which means that these significant results have to be regarded with caution. The sensitivity analyses support this conclusion. Meta regression analyses suggest that less weeks of hormonal treatment are associated with a better effect (a significant negative slope). It is possible that anorexia patients benefit on short term when it comes to (hormonal) medication, but fail to have a better recovery on long term. There are indications that for example alterations in the regulation of the hormone leptin may play a part in the persistence of anorexia nervosa. During recovery of anorexia patients, normalization of leptin levels seems to precede normalization of weight; this may be a contributing factor to the difficulties patients experience with maintaining normal weights, in this case for longer treatments [35]. Larger effect sizes for weight do not necessarily mean normalization of weight. Furthermore, weight goals differ depending on the treatment phase (acute phase or maintenance phase). In the acute phase the aim of treatment is mostly weight gain. However, weight gain alone cannot be considered a successful treatment of AN as other symptoms may continue to exist (e.g. absence of menstruation, preoccupation with body weight). Most trials focus on weight restoration; unfortunately this is only one aspect of the complex pathology of AN.

We found that antidepressants had no significant effect on weight compared to placebo in the treatment of AN. [4] found that antidepressants did not only fail to improve weight when compared to placebo, but also eating related psychopathology. However when we adjusted for publication bias the effect became higher, and significant favouring antidepressants over placebo treatment. Claudino et al. however only included subjects in the acute underweight phase, while this meta-analysis had broader criteria (acute and maintenance phase).

This meta-analysis also suggests that antipsychotics had no significant effect on weight compared to placebo in the treatment of AN. This latter is in line with the findings of Kishi et al. [9] and Lebow [10]. Kishi concludes on basis of their results that “taken together, the currently available evidence seems to tilt the risk-benefit balance against antipsychotics in patients with anorexia nervosa”.

Considering the grave consequences of AN all small steps that are helpful in treatment should be taken into consideration. However the results of this study should be interpreted within the study’s limitations.

Antidepressants and antipsychotics are the most commonly used medicamental treatments for anorexia nervosa in the Netherlands. Yet in both cases we failed to reveal efficacy when compared to placebo. There has been a lot of research trying to explain the placebo response, focusing on non/specific factors, expectancy and conditioning. Some studies have tried to identify “placebo prone personality types” with no consistent conclusions. There are some indications that use others as a healing resource and build positive relationships tend to benefit more from placebos, a hypothesis that supports continuity of care and effective interpersonal relationships to produce those placebo effects [36].

The limited findings of antidepressant and antipsychotic trials also raise an ethical dilemma: should those medications be used to treat anorexia nervosa in the acute phase? Clinicians should consider whether it is more beneficial to treat anorexia nervosa patients with pharmacotherapy in the stabilisation or prevention relapse phase, not aiming only at weight but also secondary symptoms as depression (see also the conclusion of Claudino et al. [4]). The design of the studies included also raise moral dilemmas. For example, how ethical is it to withhold treatment from anorexic patients and offer them a placebo instead? Furthermore, not all studies reported funding information. This aspect is essential when medication trials are published.

### Limitations and recommendations

Several limitations of our meta analysis caution against over-interpretation of the results. One methodological shortcoming is that the quality of the 18 included studies was not optimal and some of the studies had very small sample sizes. Six out of eight studies of hormonal therapy for example had an unclear or high risk of bias. Yet those studies yielded positive findings. The power was an important problem, we could not include the number of studies that were necessary according to power calculations, which unfortunately made it impossible to run subgroup analyses for antidepressants and antipsychotics.

The studies contain relatively small patient samples, lending the results to a narrow foundation. In addition there is major variety in treatment and patient groups. The heterogeneity in the comparison between hormonal therapy and placebo was large. Furthermore some patients in the experimental as well as control conditions received various forms of adjunctive treatment, varying from very intensive clinical treatment to weekly outpatient sessions, making it difficult to draw conclusions. Some studies included adolescents as young as 12 years old. Changes in weight could be attributed to growth and not medication, especially in longer RCT’s.

Other methodological shortcomings of the studies involve the outcome measures and reporting of outcomes. Although we've tried to report on many outcome measures and subgroups, this on the one hand reduced our sample sizes considerably, making it very hard to draw conclusions. On the other hand it lead to a lot of irrelevant results. Furthermore, the primary outcome measure was improvement of weight, as this is considered the first step in recovering from AN. Most of the trials therefore reported on weight, but unfortunately did so in various ways, making aggregation of the results very difficult, if not impossible. More over a number of studies only reported significant results, leaving out non significant but possibly relevant data and information. Because we inserted published data only, this may have caused a bias in our analyses.

Some evidence of heterogeneity was found between studies. Two main reasons for this could be that studies were found to be pursuing similar objectives (improvement in the treatment of AN) but with differences in goals and in expected actions of the pharmacological interventions.

### Recommendations

Although considerable research had been devoted to AN evidence considering treatment efficacy is scarce. As demonstrated in this MA, trials reporting on pharmacotherapy compared to placebo had major limitations. Therefore it is of extreme importance that researchers would systematically use the same outcome measures. Furthermore the use of dichotomous data or the use of categories of levels of improvement in symptoms would facilitate research in the future. There is an urgent need for more and better quality studies with a better operationalization of the terms of improvement for AN. RCT’s need to focus not only on weight restoration but on a broader definition of improvement. In these future studies, researchers must also attend to issues of statistical power and research design.

## Additional file

Additional file 1:
**PubMedSearchString.**

## Abbreviations

AN: Anorexia nervosa
BMI: Body mass index
IBW: Ideal body Weight
RCT: Randomized controlled trial
AD: Antidepressants
AH: Antihistamines
AP: Antipsychotics
CBT: Cognitive Behavioral Therapy
ch.: Change
GH: Growth hormone
GI: Gastrointestinal
H: Hormones (other)
n.m.: Not mentioned
OM: Osteoporosis medication
