# Supplementary material for: Meta analysis on the efficacy of pharmacotherapy versus placebo on anorexia nervosa
Source: J Eat Disord. 2014 Oct 30;2:27. doi: 10.1186/s40337-014-0027-x (PMC4221720; doi:10.1186/s40337-014-0027-x)
Supplement: Additional file 1: — PubMedSearchString. [file 40337_2014_27_MOESM1_ESM.docx]

Additional file 1

PubMedSearchString

**#1 Eetstoornissen (event. Therapy als SH)**

"Eating Disorders"[Mesh:NoExp] OR "Anorexia Nervosa"[Mesh] OR "Bulimia Nervosa"[Mesh] OR "eating disturbance"[tiab] OR "eating disturbed"[tiab] OR binge[tiab] OR ("eating disorder*"[tiab] OR "Anorexia"[tiab] OR "Bulimia"[tiab] NOT medline[sb])

**#2 Therapie**

"Psychotherapy"[Mesh] OR "Nutrition Therapy"[Mesh] OR "Counseling"[Mesh:NoExp] OR "Directive Counseling"[Mesh] OR "Drug Therapy"[Mesh:NoExp] OR "drug therapy "[Subheading] OR "interpersonal therapy"[tiab] OR "Systemic therapy"[tiab] OR mindfulness[tiab] OR mindfullness[tiab] OR mandometer[tiab] OR "mentalization based"[tiab] OR "mentalisation based"[tiab] OR (psychotherap*[tiab] OR therap*[tiab] OR counseling[tiab] OR counselling[tiab] NOT medline[sb])

**#3 effectiviteit (als uitkomstmaat)**

treatment outcome[mesh] OR outcome[tiab] OR effectiv*[tiab] OR efficacy[tiab]

**#4 Publicatie typen filter**

NOT ("addresses"[Publication Type] OR "biography"[Publication Type] OR "case reports"[Publication Type] OR "comment"[Publication Type] OR "directory"[Publication Type] OR "editorial"[Publication Type] OR "festschrift"[Publication Type] OR "interview"[Publication Type] OR "lectures"[Publication Type] OR "legal cases"[Publication Type] OR "legislation"[Publication Type] OR "letter"[Publication Type] OR "news"[Publication Type] OR "newspaper article"[Publication Type] OR "patient education handout"[Publication Type] OR "popular works"[Publication Type] OR "congresses"[Publication Type] OR "consensus development conference"[Publication Type] OR "consensus development conference, nih"[Publication Type] OR "practice guideline"[Publication Type])

**#5 RCT-filter**

(randomized controlled trial [pt] OR controlled clinical trial [pt] OR randomized controlled trials [mh] OR random allocation [mh] OR double-blind method [mh] OR single-blind method [mh] OR clinical trial [pt] OR clinical trials [mh] OR "clinical trial" [tw] OR ((singl* [tw] OR doubl* [tw] OR trebl* [tw] OR tripl* [tw]) AND (mask* [tw] OR blind* [tw])) OR "latin square" [tw] OR placebos [mh] OR placebo* [tw] OR random* [tw] OR research design [mh:noexp] OR comparative study [pt] OR evaluation studies [pt] OR follow-up studies [mh] OR prospective studies [mh] OR cross-over studies [mh] OR control[tw] OR controll*[tw] OR prospectiv* [tw] OR volunteer* [tw]) NOT (animal [mh] NOT human [mh])

**Aantal records met RCT-filter (AND) 02-11-2009: 1690**

| Search | |  | Result | |
| --- | --- | --- | --- | --- |
| [#16](http://www.ncbi.nlm.nih.gov/sites/?querykey=16&dbase=pubmed&querytype=eSearch&) | | Search #14 NOT #15 **deze studies mis je met het RCT-filter** | [1980](http://www.ncbi.nlm.nih.gov/sites/?cmd=HistorySearch&querykey=16&) | |
| [#15](http://www.ncbi.nlm.nih.gov/sites/?querykey=15&dbase=pubmed&querytype=eSearch&) | | Search #14 AND (randomized controlled trial [pt] OR controlled clinical trial [pt] OR randomized controlled trials [mh] OR random allocation [mh] OR double-blind method [mh] OR single-blind method [mh] OR clinical trial [pt] OR clinical trials [mh] OR "clinical trial" [tw] OR ((singl* [tw] OR doubl* [tw] OR trebl* [tw] OR tripl* [tw]) AND (mask* [tw] OR blind* [tw])) OR "latin square" [tw] OR placebos [mh] OR placebo* [tw] OR random* [tw] OR research design [mh:noexp] OR comparative study [pt] OR evaluation studies [pt] OR follow-up studies [mh] OR prospective studies [mh] OR cross-over studies [mh] OR control[tw] OR controll*[tw] OR prospectiv* [tw] OR volunteer* [tw]) NOT (animal [mh] NOT human [mh]) | [1690](http://www.ncbi.nlm.nih.gov/sites/?cmd=HistorySearch&querykey=15&) | |
| [#14](http://www.ncbi.nlm.nih.gov/sites/?querykey=14&dbase=pubmed&querytype=eSearch&) | | Search #13 NOT ("addresses"[Publication Type] OR "biography"[Publication Type] OR "case reports"[Publication Type] OR "comment"[Publication Type] OR "directory"[Publication Type] OR "editorial"[Publication Type] OR "festschrift"[Publication Type] OR "interview"[Publication Type] OR "lectures"[Publication Type] OR "legal cases"[Publication Type] OR "legislation"[Publication Type] OR "letter"[Publication Type] OR "news"[Publication Type] OR "newspaper article"[Publication Type] OR "patient education handout"[Publication Type] OR "popular works"[Publication Type] OR "congresses"[Publication Type] OR "consensus development conference"[Publication Type] OR "consensus development conference, nih"[Publication Type] OR "practice guideline"[Publication Type]) | [3675](http://www.ncbi.nlm.nih.gov/sites/?cmd=HistorySearch&querykey=14&) | |
| [#13](http://www.ncbi.nlm.nih.gov/sites/?querykey=13&dbase=pubmed&querytype=eSearch&) | | Search #11 AND #12 | [4685](http://www.ncbi.nlm.nih.gov/sites/?cmd=HistorySearch&querykey=13&) | |
| [#12](http://www.ncbi.nlm.nih.gov/sites/?querykey=12&dbase=pubmed&querytype=eSearch&) | | Search "Psychotherapy"[Mesh] OR "Nutrition Therapy"[Mesh] OR "Counseling"[Mesh:NoExp] OR "Directive Counseling"[Mesh] OR "Drug Therapy"[Mesh:NoExp] OR "drug therapy "[Subheading] OR "interpersonal therapy"[tiab] OR "Systemic therapy"[tiab] OR mindfulness[tiab] OR mindfullness[tiab] OR mandometer[tiab] OR "mentalization based"[tiab] OR "mentalisation based"[tiab] OR (psychotherap*[tiab] OR therap*[tiab] OR counseling[tiab] OR counselling[tiab] NOT medline[sb]) | [1625354](http://www.ncbi.nlm.nih.gov/sites/?cmd=HistorySearch&querykey=12&) | |
| [#11](http://www.ncbi.nlm.nih.gov/sites/?querykey=11&dbase=pubmed&querytype=eSearch&) | Search "Eating Disorders"[Mesh:NoExp] OR "Anorexia Nervosa"[Mesh] OR "Bulimia Nervosa"[Mesh] OR "eating disturbance"[tiab] OR "eating disturbed"[tiab] OR binge[tiab] OR ("eating disorder*"[tiab] OR "Anorexia"[tiab] OR "Bulimia"[tiab] NOT medline[sb]) | | 08:34:50 | [20744](http://www.ncbi.nlm.nih.gov/sites/?cmd=HistorySearch&querykey=11&) |

| **Search** | **PubMed 21-10-2009** | **Result** |
| --- | --- | --- |
| [#48](http://www.ncbi.nlm.nih.gov/sites/?querykey=48&dbase=pubmed&tab=History&querytype=eSearch&) | Search #42 NOT #46 **(dit mis je als je alleen #46 neemt t.o.v. #42)** | [2045](http://www.ncbi.nlm.nih.gov/sites/?cmd=HistorySearch&querykey=48&tab=&) |
| [#47](http://www.ncbi.nlm.nih.gov/sites/?querykey=47&dbase=pubmed&tab=History&querytype=eSearch&) | Search #46 AND (treatment outcome[mesh] OR outcome[tiab] OR effectiv*[tiab] OR efficacy[tiab]) => **mesh en [tiab] niet medline set - effectiviteit** | [650](http://www.ncbi.nlm.nih.gov/sites/?cmd=HistorySearch&querykey=47&tab=&) |
| [#46](http://www.ncbi.nlm.nih.gov/sites/?querykey=46&dbase=pubmed&tab=History&querytype=eSearch&) | Search #44 AND #45 => **set mesh en [tiab] op niet medline** | [2854](http://www.ncbi.nlm.nih.gov/sites/?cmd=HistorySearch&querykey=46&tab=&) |
| [#45](http://www.ncbi.nlm.nih.gov/sites/?querykey=45&dbase=pubmed&tab=History&querytype=eSearch&) | Search "Psychotherapy"[Mesh] OR "Nutrition Therapy"[Mesh] OR "Counseling"[Mesh:NoExp] OR "Directive Counseling"[Mesh] OR "interpersonal therapy"[tiab] OR "Systemic therapy"[tiab] OR mindfulness[tiab] OR mindfullness[tiab] OR (psychotherap*[tiab] OR therap*[tiab] OR counseling[tiab] OR counselling[tiab] NOT medline[sb]) | [283332](http://www.ncbi.nlm.nih.gov/sites/?cmd=HistorySearch&querykey=45&tab=&) |
| [#44](http://www.ncbi.nlm.nih.gov/sites/?querykey=44&dbase=pubmed&tab=History&querytype=eSearch&) | Search "Eating Disorders"[Mesh:NoExp] OR "Anorexia Nervosa"[Mesh] OR "Bulimia Nervosa"[Mesh] OR ("eating disorder"[tiab] OR "eating disorders"[tiab] OR "Anorexia Nervosa"[tiab] OR "Bulimia Nervosa"[tiab] NOT medline[sb]) | [17348](http://www.ncbi.nlm.nih.gov/sites/?cmd=HistorySearch&querykey=44&tab=&) |
| [#43](http://www.ncbi.nlm.nih.gov/sites/?querykey=43&dbase=pubmed&tab=History&querytype=eSearch&) | Search #42 AND (treatment outcome[mesh] OR outcome[tiab] OR effectiv*[tiab] OR efficacy[tiab]) => **pubmed set - effectiviteit** | [1321](http://www.ncbi.nlm.nih.gov/sites/?cmd=HistorySearch&querykey=43&tab=&) |
| [#42](http://www.ncbi.nlm.nih.gov/sites/?querykey=42&dbase=pubmed&tab=History&querytype=eSearch&) | Search #40 AND #41 => **set over heel pubmed** | [4899](http://www.ncbi.nlm.nih.gov/sites/?cmd=HistorySearch&querykey=42&tab=&) |
| [#41](http://www.ncbi.nlm.nih.gov/sites/?querykey=41&dbase=pubmed&tab=History&querytype=eSearch&) | Search "Psychotherapy"[Mesh] OR "Nutrition Therapy"[Mesh] OR "Counseling"[Mesh:NoExp] OR "Directive Counseling"[Mesh] OR "interpersonal therapy"[tiab] OR "Systemic therapy" [tiab] OR mindfulness[tiab] OR mindfullness[tiab] OR psychotherap*[tiab] OR therap*[tiab] OR counseling[tiab] OR counselling[tiab] | [1519845](http://www.ncbi.nlm.nih.gov/sites/?cmd=HistorySearch&querykey=41&tab=&) |
| [#40](http://www.ncbi.nlm.nih.gov/sites/?querykey=40&dbase=pubmed&tab=History&querytype=eSearch&) | Search "Eating Disorders"[Mesh:NoExp] OR "Anorexia Nervosa"[Mesh] OR "Bulimia Nervosa"[Mesh] OR "eating disorder"[tiab] OR "eating disorders"[tiab] OR "Anorexia Nervosa"[tiab] OR "Bulimia Nervosa"[tiab] | [20529](http://www.ncbi.nlm.nih.gov/sites/?cmd=HistorySearch&querykey=40&tab=&) |

**Kopieerblok 11-11-2009 (records: 3675):**

((("Eating Disorders"[Mesh:noexp] OR "Anorexia Nervosa"[Mesh] OR "Bulimia Nervosa"[Mesh] OR "eating disturbance"[tiab] OR "eating disturbed"[tiab] OR binge[tiab] OR ("eating disorder*"[tiab] OR "Anorexia"[tiab] OR "Bulimia"[tiab] NOT medline[sb])) AND ("Psychotherapy"[Mesh] OR "Nutrition Therapy"[Mesh] OR "Counseling"[Mesh:noexp] OR "Directive Counseling"[Mesh] OR "Drug Therapy"[Mesh:noexp] OR "drug therapy "[Subheading] OR "interpersonal therapy"[tiab] OR "Systemic therapy"[tiab] OR mindfulness[tiab] OR mindfullness[tiab] OR mandometer[tiab] OR "mentalization based"[tiab] OR "mentalisation based"[tiab] OR (psychotherap*[tiab] OR therap*[tiab] OR counseling[tiab] OR counselling[tiab] NOT medline[sb]))) NOT ("addresses"[Publication Type] OR "biography"[Publication Type] OR "case reports"[Publication Type] OR "comment"[Publication Type] OR "directory"[Publication Type] OR "editorial"[Publication Type] OR "festschrift"[Publication Type] OR "interview"[Publication Type] OR "lectures"[Publication Type] OR "legal cases"[Publication Type] OR "legislation"[Publication Type] OR "letter"[Publication Type] OR "news"[Publication Type] OR "newspaper article"[Publication Type] OR "patient education handout"[Publication Type] OR "popular works"[Publication Type] OR "congresses"[Publication Type] OR "consensus development conference"[Publication Type] OR "consensus development conference, nih"[Publication Type] OR "practice guideline"[Publication Type]))
